# Effects of temperature on feed intake and plasma chemistry after exhaustive exercise in triploid brown trout (*Salmo trutta* L)

**DOI:** 10.1007/s10695-016-0290-7

**Published:** 2016-09-13

**Authors:** Andrew C. Preston, John F. Taylor, Per Gunnar Fjelldal, Tom Hansen, Hervé Migaud

**Affiliations:** 1 Institute of Aquaculture, School of Natural Sciences, University of Stirling, Stirling, FK9 4LA UK; 2 Institute of Marine Research (IMR), Matre Research Station, 5984 Matredal, Norway

**Keywords:** Brown trout, Temperature, Triploid, Exercise, Blood chemistry, Deformity

## Abstract

The physiological effect of temperature on feed intake and haematological parameters after exhaustive swimming in diploid and triploid brown trout (*Salmo trutta*) was investigated. Trout were exposed to an incremental temperature challenge (2 °C/day) from ambient (6 °C) to either 10 or 19 °C. Feed intake profiles did not differ between ploidy at 10 °C; however, triploids had a significantly higher total feed intake at 19 °C. After 24 days, each temperature–ploidy group was exposed to exhaustive swimming for 10 min. The haematological response differed between ploidy, with the magnitude of the response affected by temperature and ploidy. Post-exercise, acid–base and ionic differences were observed. Plasma lactate increased significantly from rest for both temperature and ploidy groups, but glucose increased significantly at higher temperature. Post-exercise, triploids at 19 °C had significantly higher osmolality and cholesterol than diploids, but differences were resumed within 4 h. Elevated alkaline phosphatase (ALP) and aspartate aminotransferase (AST) in fish at higher temperature suggested greater tissue damage; however, both ploidy responded similarly. Despite no significant differences in deformity prevalence, the type and location of deformities observed differed between ploidy (decreased intervertebral space with higher prevalence in tail area and fin regions for diploids, while vertebral compression, fusion in cranial and caudal trunks for triploids). These results suggest triploids have greater appetite than diploids at elevated temperature and that triploids suffer similar blood disturbances after exercise as diploids. These findings have implications for the management of freshwater ecosystems and suggest that stocking triploid brown trout may offer an alternative to diploid brown trout.

## Introduction

The induction of triploidy has become a popular tool for the production of sterile fish in the aquaculture and fisheries sectors (Manuel et al. 2013; Taylor et al. 2014). Triploids have been commercially or experimentally produced in many fish and shellfish species by hydrostatic pressure, heat, cold or chemical shocks (Piferrer et al. 2009). Recently, the benefits of triploidy have become attractive to prevent cross-breeding between farmed and wild fish stocks and alleviate potential genetic introgression (Taranger et al. 2010; Migaud et al. 2013), but also for reported enhanced growth rates during the production cycle (Taylor et al. 2013) in salmonids. Currently, farmed all-female triploid brown trout *Salmo trutta* are being stocked into recreational freshwater fisheries within England and Wales as part of a National Trout and Grayling Strategy to reduce the risk of breeding with wild brown trout populations (Environment Agency 2009). Triploid trout are cultured using commercial aquaculture practices and then released into freshwater ecosystems to support recreational fisheries. Many fisheries endorse catch-and-release policies, and therefore triploid fish must be able to recover physiologically from strenuous exercise at a variety of temperatures (Clark et al. 2012). However, concerns have been raised for the use of triploids in wild rivers due to their increased sensitivity to fluctuating environmental conditions and exercise tolerance as part of catch-and-release fisheries. Previously, triploid Atlantic salmon subjected to a combination of high temperature (19 °C) and lowered oxygen saturation (70 %) exhibited reduced feed intake and growth and significantly higher mortality (Hansen et al. 2015). Triploids have larger nuclei as a result of the increase in genomic content, with subsequent increases in cellular volume and cell size as well as an overall reduction in cell numbers (Benfey 1999). The increase of cell size causes a reduction in the cellular surface-to-volume ratio which could alter transport processes across cell membranes (Maxime 2008). In addition, exhaustive exercise imposed by capture stressors has been shown to impact on the ion, acid–base and metabolic status of teleosts (Wilkie et al. 1997; Meka and McCormick 2005; Gale et al. 2011; Gingerich and Suski 2012). Previous studies have found that the thermal tolerance of triploids is similar to diploids, as indicated by the time taken to reach critical thermal maxima (CTM) of brook trout (*Salvelinus fontinalis*) and rainbow trout (*Oncorhynchus mykiss*) (Benfey et al. 1997; Galbreath et al. 2006). On the other hand, routine metabolic rates differ between diploid and triploid brook trout and Atlantic salmon (*Salmo salar*), with triploids having higher metabolic rates than diploids at lower temperatures, and lower metabolic rates than diploids at higher temperatures (Atkins and Benfey 2008).

The thermal tolerance of triploid fish has been investigated, and results showed increased mortality at high temperatures (Ojolick et al. 1995). Triploid brown trout reared in seawater for 12 weeks showed a cumulative mortality of 51 % at 18 °C versus 3 % at 14 °C (Altimiras et al. 2002). Similarly, Hyndman et al. (2003b) found that diploid brook trout recovered from exhaustive exercise at 19 °C, whereas 90 % of triploids died within 4 h of exhaustive exercise at this temperature. It was suggested that the magnitude of the physiological disturbance was greater in triploid trout, which resulted in failure to restore muscle metabolites back to pre-exercise conditions, causing significant mortality. Water temperature may also affect cardiac response, and differences in heart morphology between ploidy have been observed (Fraser et al. 2013). In rainbow trout, heart rate was similar between ploidy at 10 °C; however, at higher temperatures, more triploid trout suffered from cardiac arrhythmia than diploids (Verhille et al. 2013). This suggests that the reduced tolerance to high temperature in triploid rainbow trout may be due to reduced cardiovascular scope and oxygen delivery (Verhille et al. 2013). Furthermore, triploid fish are also known to be more prone to skeletal deformities (Leclercq et al. 2011; Taylor et al. 2013), and it has been shown that spinal malformation affects swimming capacity and recovery in triploid Atlantic salmon (Powell et al. 2009). It is important to establish whether the same is true in brown trout.

The objectives of the present study were (1) to determine the effects of acute temperature increase on feed intake, (2) investigate the changes to the biochemical profile before and after exhaustive exercise (simulated catch-and-release scenario) at ecologically relevant temperatures, and (3) determine whether deformity prevalence differed between ploidy groups.

## Materials and methods

### Fish stock and rearing conditions

Experimental work was undertaken at the Institute of Marine Research Station, Matredal, Western Norway. In November 2011, eggs from eight female brown trout were pooled into one egg batch and fertilized with pooled milt from three males. The ova and sperm were obtained from a commercial production site for portion size brown trout in Tyssedal, Norway. The trout originated from the Tunhovd Lake in Eastern Norway and had been in culture for ten generations. After fertilization, eggs were split into two batches and one batch was subjected to a hydrostatic pressure shock of 655 bar applied for 6 min 15 s at 37 min 30 s post-fertilization at 8 °C (TRC-APV, Aqua Pressure Vessel, TRC Hydraulics inc., Dieppe, Canada), giving two ploidy groups with approximately 3500 eggs each. Each ploidy group was incubated discretely in a single incubation tray in a flow-through system at 7 °C. Eggs were mechanically agitated to allow dead eggs to be separated from live eggs at the eyed-egg stage (~200 days). First-feeding fry from each incubator were randomly distributed into fibreglass tanks (1 × 1 × 0.5 m) under continuous light and at 10 °C (3 replicates/ploidy; 400 fish/replicate tank). The numbers of fish in each tank were then reduced to 300 in order to maintain optimal stocking density. Ambient water temperature decreased gradually to 6 °C by December 2012 and remained in the range of 5.9–6.4 °C until the start of the feeding study. Ploidy status was determined by randomly blood sampling 85 first-feeding fish per ploidy and measuring red blood cell nuclear diameters according to previously published protocol (Preston et al. 2013). All fish screened were shown to conform to the correct ploidy, indicating 100 % triploid rate.

### Feed intake study

Fish were reared in six freshwater tanks (3/ploidy, 300 fish/tank) until mid-December 2012, when all fish were measured for length and weight, then 320 fish per ploidy were randomly distributed into sixteen freshwater tanks with 40 fish/tank (1 × 1 × 0.6 m, 0.6 m^3^), giving eight tanks with diploids (mean weight 70.53 ± 1.02 g; length 164.94 ± 0.74 mm) and eight tanks with triploids (mean weight 79.87 ± 1.18 g; length 173.72 ± 0.80 mm). All tanks were kept at ambient water temperature for 48 h before being subjected to a thermal challenge where temperature was increased from ambient (6.5 ± 0.5 °C) to 10 ± 0.8 °C or 19 ± 0.9 °C, respectively, in 2 °C day^−1^ increments, producing two temperature regimes (10 or 19 °C, with two ploidy groups (diploid and triploid) per temperature group and 4 replicates each). The temperatures used (10 and 19 °C) within this study were chosen after reviewing river temperature data provided by the Environment Agency (data not shown) and represented the lower and upper temperatures brown trout are exposed to post-release into freshwater fisheries in England. All tanks were fed three meals per day using ArvoTec 2000 drum feeders and a commercially available 3-mm dry feed (Nutra parr, Skretting, Norway) for a period of 24 days. Meals consisting of 100 g of dry pellet were dispensed into the tanks between 0800 and 0900 am (meal 1), 1130–1230 (meal 2) and 1400–1500 pm (meal 3) each day. Waste feed was collected 25 min after each meal, excess water was drained off, and feed weighed according to the method described by Helland et al. (1996) and used to calculate the dry weight of feed eaten according to a recovery coefficient (hereafter called feed intake). Fulton’s condition factor (K) was calculated as *K* = (100 W)/FL^3^, where *W* is whole body mass (g) and FL is fork length (mm). Specific growth rate (SGR) over the experimental period was calculated as SGR = 100 (e^g^ − 1), where *g* = (ln*W*_f_ − lnW_i_)/(*t*_f_ − *t*_i_), *W*_f_ and *W*_i_ are the mean final and initial *W*, respectively, and (*t*_f_ − *t*_i_) is the duration of the experimental period in days. The mean growth rate was also measured as the thermal growth coefficient (TGC) to account for any difference between ploidy groups as follows: TGC = (*W*_f_^1/3^ − *W*_i_^1/3^) × (1000/DD), where *W*_f_ and *W*_i_ are as previously addressed for SGR and DD is the cumulative daily water temperature (°C).

### Recovery from exhaustive exercise

Following the feed intake study, fish (each ploidy and temperature group) were starved for a period of 48 h prior to being exposed to an exhaustive exercise protocol previously described (Donaldson et al. 2010; Clark et al. 2012). We used a combination of water inflow and manual chasing for 10 min until all individuals were unable to continue burst-type swimming. A water vortex was generated by increasing the volume of water which generated a whirlpool within each tank. Oxygen was never a limiting factor during any of the experiments (≥90 % saturation).

### Sampling procedure

Following 48 h of starvation, blood samples (resting) were taken prior to exhaustive exercise with 5 fish/tank randomly sampled (*n* = 80). Thereafter, using the same protocol, blood sampling was conducted at 1, 4 and 24 h post-exhaustive exercise. Fish were rapidly caught and placed into a lethal dose of MS-222 (200 mg/L, Sigma-Aldrich, Poole, UK) and then euthanized by cranial concussion. Thereafter, weight and length were measured for each fish before blood was withdrawn from the caudal vein using a 1-ml heparinized syringe and 23 G sterile hypodermic needle. Whole blood taken was placed into a 1.5-mL microfuge tube and stored on ice before being centrifuged at 9676×*g* for 3 min. The resulting plasma was aliquoted into two micro-Eppendorfs and snap-frozen using liquid nitrogen before storage at −70 °C for biochemical analyses. This process was achieved in less than 5 min for all samples taken to prevent changes that may occur in blood chemistry post-killing. The work was conducted in accordance with the laws and regulations controlling experiments and procedures on live animals in Norway following the Norwegian Regulation on Animal Experimentation 1996 and reviewed by the University of Stirling Animal Welfare and Ethical Review Body.

### Blood chemistry analysis

Plasma ions (Na^+^, K^+^ and Cl^−^) were analysed by ion-selective electrodes (ISE) using standard kits for the COBAS c111 auto-analyser (Roche Diagnostics, Indianapolis, USA). For all other plasma parameters (lactate, glucose, pH, phosphorus, calcium, magnesium, potassium, triglycerides, cholesterol, alkaline phosphatase and aspartate aminotransferase), the Maxmat (PL II) multidisciplinary diagnostic enzyme-linked immunosorbent assay (ELISA) analyser was used and calibrated before use on each day. Plasma pH was determined using a micro-pH meter (Thermo Scientific Orion). Total plasma osmolality was measured using freezing point determination (Fiske micro-osmometer Model 210, Norwood, MA, USA).

### Radiography and vertebral deformities

After blood sampling, diploid and triploid brown trout from each temperature (20 fish/tank, *n* = 160/ploidy) were individually labelled, stored on ice prior to being frozen on a flat surface at −20 °C for later radiography. Radiographs were taken using a portable X-ray apparatus (HI-Ray 100, Eickenmeyer Medizintechnik für Tierärzte e.K., Tuttlingen, Germany) and 30 × 40 cm sheet film (FUJIFILM IX 50, FUJIFILM Corp., Tokyo, Japan). The film was exposed at 12 mA s and 44 kV and developed using a manual developer (Cofar Cemat C56D, Arcore (MI), Italy) with Kodak Professional manual fixer and developer (KODAK S.A., Paris, France). The pictures were digitized by scanning (Epson Expression 10,000 XL, Seiko Epson Corp., Nagano-Ken, Japan). The vertebral column of each fish was examined using Adobe Photoshop CS2, and the vertebral column was divided into 4 regions (R) according to Kacem et al. (1998): R1 (cranial trunk, V1 → V8), R2 (caudal trunk, V9 → V30), R3 (tail, V31 → V49) and R4 (tail fin, V50 → V57/58/59). The number of vertebrae (*V*) per fish, the location and type of deformity were determined according to Witten et al. (2009).

### Statistical analysis

Comparison of the response variable feed intake was made using a two-way general linear model (GLM) with the independent variables of temperature and ploidy, and their interaction. Comparison of the physiological response variables was made using a three-way mixed model GLM with the independent variables of time, temperature and ploidy as well as their two- and three-way interaction. Post hoc testing was determined using Tukey’s multiple comparison tests to separate significant means where appropriate. Data were checked for normality and homogeneity of variance following Kolmogorov–Smirnov and Levene’s tests and examination of residual plots. Feed intake data (% biomass) and deformity rate were arcsine-transformed prior to analysis, and plasma parameter data which did not conform to normality were subsequently log-transformed prior to analyses. Differences in vertebrae number and deformity rate were tested using nonparametric Kruskal–Wallis test. All statistical tests were performed using Minitab version 16.1 with a significance level of *P* < 0.05. All results are presented as mean ± SEM.

## Results

### Feed intake and growth

**Fig. 1:**
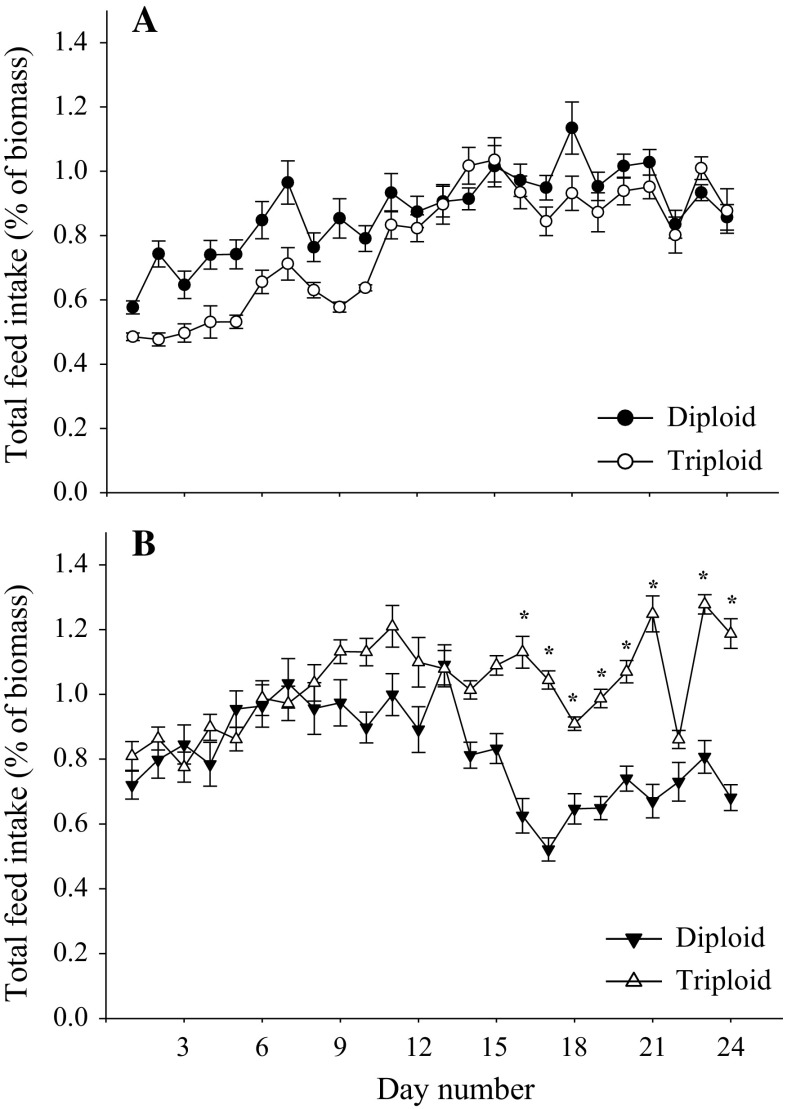
Feed intake profiles of diploid and triploid brown trout (*Salmo trutta* L.) during the 24-day trial period. All trout were reared in quadruplicate 455-L freshwater tanks at 6 °C, then acclimated to 10 and 19 °C (2 °C increment/day), then fed to satiation three times daily. Total feed intake per day for 10 °C (**a**) and 19 °C (**b**) is the sum of meals 1–3 each day. *Asterisk* indicates a significant difference between ploidy (GLM, *P* < 0.05)

The total daily feed intake at 10 °C was not significantly different between ploidy at any time point (Fig. 1a). At 19 °C, the total feed intake was significantly higher in triploids than in diploids from day 16 to 24 except at day 22 (Fig. 1b). Prior to the start of the experiment, triploids had a significantly higher body mass and fork length and lower condition factor than diploids (Table 1a). At the end of the trial, triploids remained heavier, longer in length and had lower condition factors than diploids. SGR and TGC did not vary significantly between ploidy at any time point for both temperature groups; however, TGC values were significantly reduced in fish reared at 19 °C (Table 1b).

**Table 1:** Growth performance of diploid and triploid brown trout at the start of the experiment (A), and after the swimming protocol (B)

Treatment		A. Exp. start			
Temperature (°C)	Ploidy	*N*	*W* (g)	FL (mm)	*K*
10	Diploid	80	70.1 ± 3.1^a^	165.0 ± 2.2^a^	1.54 ± 0.01^a^
10	Triploid	80	80.3 ± 3.5^b^	174.1 ± 2.4^b^	1.53 ± 0.01^b^
10	Diploid	80	70.1 ± 0.2^a^	164.8 ± 2.0^a^	1.54 ± 0.01^a^
19	Triploid	80	79.3 ± 3.5^b^	173.5 ± 2.3^b^	1.49 ± 0.01^b^

Treatment		B. Post-swimming protocol					
Temperature (°C)	Ploidy	*N*	*W* (g)	FL (mm)	*K*	SGR	TGC
10	Diploid	80	81.4 ± 3.6^b,c^	180.2 ± 2.5^b^	1.36 ± 0.01^a^	0.30 ± 0.03^a^	0.46 ± 0.04^a^
10	Triploid	80	91.3 ± 4.1^a^	189.4 ± 2.5^a^	1.31 ± 0.01^b^	0.27 ± 0.02^a^	0.40 ± 0.07^a^
10	Diploid	80	76.0 ± 3.0^c^	178.2 ± 2.1^b^	1.32 ± 0.01^b^	0.18 ± 0.06^a^	0.15 ± 0.05^b^
19	Triploid	80	87.1 ± 3.9^a,b^	187.0 ± 2.5^a^	1.30 ± 0.01^b^	0.21 ± 0.01^a^	0.18 ± 0.01^b^

Prior to the start of the experiment 80/ploidy/temperature were sampled for whole body weight (W), fork length (L) and Fulton’s condition factor (K). At sampling point b specific growth rates (SGR) and thermal growth coefficients (TGC) were determined using (*n* = 80/ploidy/temperature), and no mortality was observed in any treatment. Different superscripts denote significant differences between treatment groups (ANOVA, *P* < 0.05)

### Blood chemistry

**Fig. 2:**
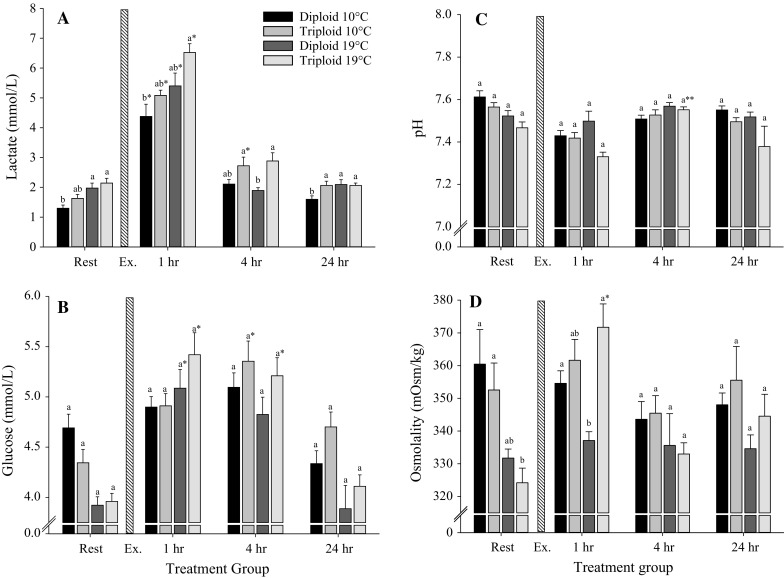
The effect of temperature, ploidy and exhaustive exercise on blood haematology of diploid and triploid brown trout for **a** lactate, **b** glucose, **c** pH and **d** osmolality levels. Pre-exercise samples are represented at rest. The *vertical stripped bar* represents the period of 10-min exercise (Ex.). After the exercise protocol, blood samples were collected at time 1, 4 and 24 h post-exercise. At each time point, 20 fish/ploidy/temperature were sampled and expressed as mean ± SE. *Different letters* indicate significant differences between ploidy at a given time point. *Single asterisk* indicates a significant difference to resting levels, and *double asterisk* indicates a significant difference between 1 and 4 h post-exercise within a given ploidy–temperature regime (GLM, *P* < 0.05)

Lactate was significantly elevated from resting at 1 h post-exercise in all groups (GLM, *F* = 116.14, *P* < 0.001) with significantly higher levels in triploid 19 °C compared to diploid 10 °C (GLM, *F* = 27.08, *P* < 0.001) (Fig. 2a). At 4 h post-exercise, lactate returned to resting levels in all groups, except for triploid 10 °C which remained significantly elevated from resting (GLM, *F* = 15.20, *P* < 0.001). All groups returned to resting by 24 h post-exercise. Significant effects of the interaction between time and temperature were found (GLM, *F* = 3.02, *P* = 0.042). An example of the GLM outputs corresponding to the lactate data is presented in Table 2.

**Table 2:** A typical example of a three-way GLM performed on lactate data using the method of sequential sums of square for test

Source (lactate)	*DF*	Seq SS	Adj SS	Adj MS	*F*	*P*
Time	3	10.9	10.9	3.36	116.4	0.001
Ploidy	1	0.8	0.8	0.84	27.08	0.001
Temperature	1	0.4	0.5	0.47	15.2	0.001
Time × ploidy	3	0.1	0.1	0.03	1.17	0.334
Time × temperature	3	0.28	0.3	0.09	3.02	0.042
Ploidy × temperature	1	0.01	0.0	0.01	0.32	0.573
Replicate (ploidy × temperature)	12	0.3	0.3	0.02	0.93	0.532
Time × ploidy × temperature	3	0.08	0.1	0.02	0.95	0.426
Error	36	1.1	1.1	0.03		
Total	63	14.1				

The fixed factors within the model were time, ploidy (2 or 3 N) and temperature with replicate tank nested within temperature–ploidy grouping

*DF* degrees of freedom, *Seq.SS* sequential sums of squares, *Adj MS* adjusted sequential mean squares, *F* variance, *P* probability

Temporal effects were observed at 19 °C with diploid and triploid showing significantly elevated glucose levels from resting at 1 h post-exercise (GLM, *F* = 7.97, *P* = 0.008). At 4 h post-exercise, glucose remained significantly elevated from resting levels in triploids for both temperature groups (GLM, *F* = 28.51, *P* < 0.001). Significant effects of the interaction between time and temperature were found (GLM, F = 5.04, *P* = 0.005) (Fig. 2b).

No ploidy or temperature effects were observed on plasma pH at any given time point (Fig. 2c). However, a significant effect of ploidy (GLM, *F* = 11.14, *P* = 0.002) and temperature (GLM, *F* = 4.38, *P* = 0.043) was observed from resting to 1 h post-exercise between diploid 10 and triploid 19. Plasma pH values increased significantly between 1 and 4 h post-exercise in triploid 19 °C (GLM, *F* = 6.89, *P* = 0.001) and returned to resting in all groups at 24 h post-exercise.

Resting osmolality was significantly lower in triploid 19 °C than in diploid 10 °C group (GLM, *F* = 19.55, *P* < 0.001, Fig. 2d). At 1 h post-exercise, triploid 19 °C showed significantly elevated osmolality values from resting (GLM, *F* = 4.74, *P* = 0.007) and was also significantly elevated from diploid 19 °C at both 1 and 4 h post-exercise by the interaction between time and ploidy (GLM, *F* = 5.03, *P* < 0.001). At 4 h post-exercise, plasma osmolality levels had returned to resting levels and there were no significant differences between ploidy groups (GLM, *F* = 3.49, *P* = 0.07). Significant effects of the interaction between time and temperature were found (GLM, *F* = 3.81, *P* = 0.018).

**Fig. 3:**
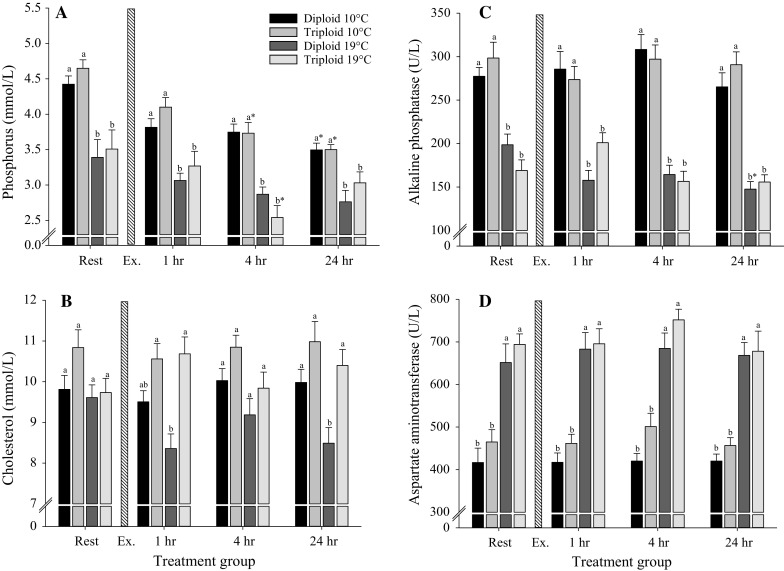
The effect of temperature, ploidy and exhaustive exercise on blood haematology of diploid and triploid brown trout for **a** phosphorus, **b** cholesterol, **c** alkaline phosphatase and **d** aspartate aminotransferase levels. Pre-exercise samples are represented at rest. The *vertical stripped bar* represents the period of 10-min exercise (Ex.). After the exercise protocol, blood samples were collected at time 1, 4 and 24 h post-exercise. At each time point, 20 fish/ploidy/temperature were sampled and expressed as mean ± SE. *Different letters* indicate significant differences between ploidy at a given time point. *Asterisk* indicates a significant difference to resting levels within a given ploidy–temperature regime (GLM, *P* < 0.05)

Pre-exercise phosphorus levels were significantly lower in fish reared at 19 °C compared to 10 °C (GLM, *F* = 123.94, *P* < 0.001, Fig. 3a) with no differences between ploidy, and this was maintained throughout the trial. In addition, triploids from both temperature regimes showed significantly reduced *P* levels at 4 h post-exercise relative to resting levels and also at 24 h for both ploidy 10 °C (GLM, *F* = 21.58, *P* < 0.001).

Plasma calcium levels were not significantly different between ploidy and temperature at any given time point (GLM, *F* = 1.30, *P* = 0.261, Table 3). However, Ca levels increased at 1 h post-exercise in both ploidy groups at 19 °C (GLM, *F* = 27.50, *P* < 0.001). Significant effects of the interaction between time and temperature were found (GLM, *F* = 4.72, *P* = 0.007). Thereafter, calcium levels returned to resting levels in all groups.

**Table 3:** The effect of temperature, ploidy and exhaustive exercise on blood haematology of diploid and triploid brown

Time (h)	Treatment	Plasma parameter					
		Calcium (mg/L)	Chloride (mmol/L)	Magnesium (mmol/L)	Potassium (mmol/L)	Sodium (mmol/L)	Triglycerides (mmol/L)
Resting	Diploid 10	96.11 ± 6.68^a^	123.03 ± 0.77^a^	0.77 ± 0.02^a^	3.43 ± 0.18^a^	152.63 ± 2.22^a^	2.24 ± 0.07^a^
Resting	Triploid 10	103.35 ± 1.38^a^	124.61 ± 0.56^a^	0.81 ± 0.02^a^	3.67 ± 0.16^a^	156.87 ± 0.50^a^	2.34 ± 0.10^a^
Resting	Diploid 19	105.33 ± 1.61^a^	122.87 ± 1.01^a^	0.81 ± 0.05^a^	4.27 ± 0.20^a^	154.41 ± 0.72^a^	1.99 ± 0.14^a^
Resting	Triploid 19	103.02 ± 0.97^a^	124.73 ± 0.82^a^	0.86 ± 0.01^a^	4.83 ± 0.35^a^	154.18 ± 0.46^a^	1.86 ± 0.11^a^
1 h	Diploid 10	114.25 ± 2.15^a^	128.13 ± 0.70^a^	0.93 ± 0.09^a^	3.80 ± 0.40^a^	159.85 ± 0.85^a^	1.72 ± 0.05^a,^*
1 h	Triploid 10	114.16 ± 1.36^a^	127.66 ± 0.74^a^	0.84 ± 0.02^a^	3.92 ± 0.20^a^	159.83 ± 0.74^a^	1.82 ± 0.08^a^*
1 h	Diploid 19	119.83 ± 4.36^a,^*	127.34 ± 0.73^a^	0.91 ± 0.03^a^	3.56 ± 0.16^a^	158.73 ± 0.91^a^	2.05 ± 0.10^a^
1 h	Triploid 19	128.30 ± 3.37^a^*	125.17 ± 0.70^a^	1.09 ± 0.12^a^	3.85 ± 0.12^a^	159.69 ± 1.10^a^	1.83 ± 0.07^a^
4 h	Diploid 10	106.18 ± 1.08^a^	125.19 ± 0.94^a^	0.81 ± 0.02^a^	3.20 ± 0.26^a^	155.89 ± 0.87^a^	1.82 ± 0.08^a^
4 h	Triploid 10	105.72 ± 1.35^a^	124.11 ± 0.65^a^	0.76 ± 0.02^a^	3.68 ± 0.15^a^	154.19 ± 0.66^a^	1.71 ± 0.10^a^
4 h	Diploid 19	106.58 ± 1.43^a^	124.15 ± 0.54^a^	0.79 ± 0.03^a^	3.25 ± 0.15^a^	153.14 ± 0.66^a^	1.80 ± 0.08^a^
4 h	Triploid 19	111.46 ± 2.18^a^	124.05 ± 1.23^a^	1.00 ± 0.19^a^	3.44 ± 0.17^a^	151.50 ± 1.82^a^	1.83 ± 0.07^a^
24 h	Diploid 10	110.96 ± 4.88^a^	127.46 ± 0.78^a^	0.79 ± 0.02^a^	3.35 ± 0.19^a^	155.96 ± 0.62^a^	1.67 ± 0.10^a^
24 h	Triploid 10	106.23 ± 1.29^a^	129.07 ± 0.77^a^	0.71 ± 0.05^a^	3.52 ± 0.18^a^	156.64 ± 0.72^a^	1.72 ± 0.07^a^
24 h	Diploid 19	111.21 ± 1.49^a^	126.30 ± 0.76^a^	0.83 ± 0.02^a^	3.31 ± 0.22^a^	154.54 ± 0.67^a^	2.03 ± 0.15^a^
24 h	Triploid 19	111.26 ± 1.37^a^	126.73 ± 0.67^a^	0.88 ± 0.03^a^	3.55 ± 0.17^a^	155.43 ± 0.49^a^	1.88 ± 0.08^a^

Pre-exercise values are represented at resting. After the exercise protocol, blood samples were collected at time 1, 4 and 24 h post-exercise. At all-time points, 20 fish/ploidy/temperature were sampled (mean ± SE)

Different letters indicate significant differences between ploidy at a given time point. * Indicates a significant difference to resting levels within a given ploidy–temperature regime (GLM, *P* < 0.05)

Plasma magnesium and potassium levels did not differ between ploidy and temperature groups with time post-exercise. Plasma sodium and chloride levels did not differ between ploidy and temperature groups with time post-exercise (Table 3).

No ploidy or temperature effects were observed on plasma triglycerides (TG) at any given time point. At 1 h post-exercise, a significant decrease relative to resting levels in both ploidy at 10 °C groups was observed (GLM, *F* = 20.55, *P* < 0.001). TG then returned to resting levels by 4 h post-exercise in all groups (Table 3).

Plasma cholesterol levels were significantly lower in diploid 19 °C compared to triploids reared at 1 h post-exercise (GLM, *F* = 28.14, *P* < 0.001, Fig. 3b). At 24 h post-exercise, levels in diploid 19 °C were significantly reduced compared to all other treatments (GLM, *F* = 20.55, *P* < 0.001).

For both ALP and AST, levels were significantly different throughout the trial in fish reared at 10 °C compared to 19 °C (GLM, *F* = 506.72, *P* < 0.001 and *F* = 265.30, *P* < 0.001, respectively, Fig. 3c, d). Significant effects of the interaction between time, ploidy and temperature on plasma ALP levels were found (GLM, *F* = 5.08, *P* = 0.005, Fig. 3c).

### Vertebral abnormalities

Vertebrae counts for diploid trout (58.1 ± 0.6) were not significantly different to their triploid siblings (58.0 ± 0.6, Kruskal–Wallis test *H* = 0.18; *DF* = 1; *P* = 0.673). Diploids had fewer fish (7 %) with radiologically deformed vertebrae (dv1+) than triploids (14 %) (Table 4). Both ploidy groups showed few individuals (<1.9 %) with severe numbers of deformed vertebrae (dv > 10). X-ray radiography revealed that in diploids the greatest prevalence of deformed vertebrae was in the tail area region (R3) and tail fin region (R4), while triploids also showed elevated deformity in cranial trunk (R1) and caudal trunk (R2) (Table 5). In diploids, v55 and v56 were the most affected vertebrae with a prevalence of 7.1 %, while in triploids *v*2 was the most affected with a prevalence of 6.6 %. Out of 20 possible classifications of deformity type based on Witten et al. (2009), seven pathology types were observed in diploids and nine in triploids (Table 5). In diploids, 72 % of all observed deformities were associated with decreased intervertebral space, principally located in *R*3 and *R*4. In triploids, decreased intervertebral space; compression and decreased intervertebral space; compression without x structure; one-sided compression; compression and fusion and widely spaced and undersized accounted for 88 % of all pathologies observed.

**Table 4:** Observed severity of deformed vertebrae in diploid and triploid brown trout characterized by radiology (*n* = 160 fish radiographed/ploidy)

Severity	No. dV	Diploid	Triploid
No deformity	0	93.13	86.25
Mild	1–5	5.63	10.63
Moderate	6–10	1.25	1.25
Severe	>10	1.25	1.88

Values are expressed as the total number of individuals (%) within each ploidy grouped according to the total number of deformed vertebrae present (no. dV = number of deformity)

**Table 5:** Observed prevalence, type and localization of vertebra deformity in diploid and triploid brown trout characterized by radiology

	Diploid					Triploid				
	R1	R2	R3	R4	Total	R1	R2	R3	R4	Total
*Type of vertebral deformity (%)*										
Decreased interV. space	3	12	**43**	14	72.3	0	4	**10**	0	14.3
Homogeneous compression	**5**	0	0	0	4.6	**2**	0	0	0	2.2
Compression and decreased interV. space	0	0	0	2	1.5	**11**	3	0	0	14.3
Compression without × structure	–	–	–	–	–	4	**8**	0	1	13.2
One-sided compression	0	0	0	**8**	7.7	2	**4**	0	**4**	11.0
Compression and fusion	0	0	0	**3**	3.1	3	**7**	0	4	14.3
Complete fusion	0	0	0	**9**	9.2	2	0	0	**7**	8.8
Widely spaced and undersized	–	–	–	–	–	9	**12**	0	0	20.9
Internal dorsal or ventral shift	0	0	0	**2**	1.5	0	0	0	**1**	1.1
Total (%)	8	12	43	37	100.0	34	38	10	18	100.0
Total (*n*)	5	8	28	24	65	31	35	9	16	91

Values are expressed as percentage of the total number of deformed vertebrae observed within each ploidy with *n* = 160 fish radiographed/ploidy. The different regions of the vertebral column are shown as follows: R1 (cranial trunk, V1 → V8), R2 (caudal trunk, V9 → V30), R3 (tail, V31 → V49) and R4 (tail fin, V50 → V59) with *R* region, *V* vertebrae and *InterV* intervertebral. For each ploidy group, the two main types of deformity within each area or within the whole vertebral column are highlighted in bold

## Discussion

Evaluation of feed intake and biochemical profiles in diploid and triploid individuals is an important aspect of evaluating physiological performance for utilization within freshwater fisheries. These studies have relevance not only to fisheries, but also to aquaculture in which triploids are used to improve fish production (Taranger et al. 2010; Taylor et al. 2015). In the present study, the temporal variation in feed intake at ecologically relevant temperatures was investigated. Indeed, at lower temperature feed intake profiles and growth were not significantly different between ploidy, supporting previously published data on Atlantic salmon (Taylor et al. 2013). Regarding temperature, our data clearly showed a gradual decline in total feed intake when diploid fish were exposed to higher temperature. Similarly, Atlantic salmon displayed a reduction in overall feeding and growth rate when fish were reared at 18 °C for 3 months (Kullgren et al. 2013). However, no such decline in feed intake was observed in triploids, suggesting that triploid brown trout can feed equally or better than diploid brown trout at high temperature and that previously observed higher mortalities in triploid brown trout reared at high temperature may be due to other environmental factors such as reduced oxygen saturation or altered water chemistry (Altimiras et al. 2002). The present results would suggest triploids might need to forage more than diploids at high temperature, which may increase fisheries capture and predation. Previously, no differences in surface feeding response were observed between diploid and triploid brown trout within an experimental stream (Preston et al. 2014). In addition, Hansen et al. (2015) indicated that triploid Atlantic salmon feed intake was significantly increased over diploids (days 11–18) when temperature was increased from 10 to 19 °C, albeit that O_2_ saturation remained ~100 % during this period. Indeed, the reverse was observed when fish were kept at 19 °C and at two differing oxygen levels (70 and 100 % O_2_ saturation) with feed intake significantly reduced when triploids were kept at 70 % O_2_ saturation. This suggests that the combination of high temperature and low O_2_ saturation induces a lower aerobic scope in triploid salmon and future studies should examine whether this biological limitation exists in triploid brown trout.

Determining the basic plasma chemistry biomarkers associated with stress response, lipid metabolism and nutritional status allows an insight into how diploid–triploid brown trout respond to temperature and recovery from a simulated fisheries capture under ecologically relevant temperatures. In this study, we used a combination of tank vortex and manual chasing to elicit burst-type swimming associated with a rod and reel capture. Resting plasma lactate and glucose concentrations did not differ between ploidy at both temperatures tested, and values were comparable to those reported from other studies (Kieffer et al. 1994; Sadler et al. 2000; Hyndman et al. 2003a). Post-exercise, elevated plasma lactate and glucose concentrations were observed in both temperature regimes, indicative of secondary metabolic response to exhaustive exercise (Barton 2002; Portz et al. 2006; Li et al. 2011). The elevation of circulating plasma glucose suggests an increase in stress hormones such as catecholamines and cortisol, not assessed in the current study, which would speed the mobilization of glucose to support increased energy demands during increased exercise (Clarkson et al. 2005). Lactate levels measured post-exercise, and clearance, were similar in both ploidy groups for a given temperature. This is contrasting with previous studies, which suggested that lactate clearance was quicker in triploid than diploid brook trout (Hyndman et al. 2003a). Plasma pH was also not significantly different between ploidy at resting or post-exercise in either temperature regimes. In previous studies, blood pH following fishing capture was shown to be reduced to 7.2 in blue marlin *Makaira nigricans* (Dobson et al. 1986), 7.5 in tuna *Katsuwonus pelamis* (Perry et al. 1985) and 7.4 in rainbow trout *Onchorhynchus mykiss* (Turner et al. 1983). In other species such as yellowfin tuna (*Thunnus albacares*), which require high rates of O_2_ delivery to tissues after exhaustive exercise, plasma pH was shown to be reduced by as much as 0.4 pH units (Lowe et al. 1998). Despite the lack of clear differences in plasma pH in the current study, relatively small deviations in pH have been shown to exhibit large effects on the oxygen-carrying capacity of the blood (Brauner and Randall 1996).

Exhaustive exercise may also disrupt ionic and osmotic balances within the blood and tissues by affecting water and ion uptake or other transport mechanisms associated with stress (Wendelaar-Bonga 1997; Gingerich and Suski 2012). In our study, a significant increase in calcium in diploid and triploids at 19 °C and plasma osmolality in triploids at 19 °C was observed, which may suggest an osmoregulatory disturbance due to the accumulation of metabolites in the muscle. However, based on previous data, these values appeared to remain within the range previously reported for salmonids (Clark et al. 2012). A previous study in sockeye salmon (*Oncorhynchus nerka*) showed similar increased plasma sodium and chloride concentrations within 30 min following a simulated capture event (Gale et al. 2011).

Diploid and triploid brown trout showed similar resting plasma cholesterol and triglyceride concentrations irrespective of temperature. In Arctic charr (*Salvelinus alpinus*), similar differences in plasma cholesterol concentrations were found between GH transgenics and control, with transgenic fish having reduced plasma triglyceride concentrations (Krasnov et al. 1999). In addition to glucose, the oxidative metabolism of lipid substrates provides energy for physiological functioning and these are efficient energy stores due to their high caloric content (Sheridan 1988). In the present study, plasma cholesterol levels in triploid brown trout were not significantly different from resting levels both pre- and post-exercise; however, temperature-dependent acute effects of exercise were observed at 1 h post-exercise at 19 °C in diploids. On the other hand, plasma triglycerides were significantly reduced in both ploidy groups at 10 °C 1 h post-exercise, however, returned to resting levels by 4 h post-exercise, indicating similar release of lipid in both ploidy groups post-exercise. It has been shown that circulating lipids originate from three sources: newly absorbed from food, recently processed in the liver and then transported to the blood or mobilized from storage sites (Sheridan 1988). In our study, all fish were starved for 48 h prior to exhaustive exercise; therefore, the experimental fish would have to utilize the oxidation of lipid released from muscle and adipose tissue to fuel the recovery process (Wang et al. 1994; Keins and Richter 1998; Richards et al. 2002; Li et al. 2011). In addition, this suggests that both ploidy mobilize similar lipids to meet the increase in energy demand from the exhaustive exercise and that similar esterification of non-esterified fatty acids into triglyceride occurred in the blood (Li et al. 2011). However, further work on muscle metabolism to determine the precise role of lipids in triploid brown trout is required.

In the present study, temperature influenced ALP and AST, but not ploidy nor exercise. Higher AST activity at 19 °C may indicate increased leakage of enzymes across damaged plasma membranes and/or increased hepatic enzyme production by diploid and triploid trout at this temperature as suggested for sea bass *Dicentrarchus labrax* (Šegvic-Bubic et al. 2013). ALP and AST activity has been shown to increase significantly after transport stress in common carp (*Cyprinus carpio*) (Dobšíková et al. 2009). In addition, ALP activity is influenced by many factors including feed intake (Sauer and Haider 1979; Congleton and Wagner 2006), temperature (Lie et al. 1988), stress transport (Dobšíková et al. 2009), life stage (Johnston et al. 1994) and diet especially phosphorus metabolism (Shao et al. 2008; Šegvic-Bubic et al. 2013). In addition, bone health issues can be influenced by ploidy (Fjelldal and Hansen 2010) and rearing temperature (Grini et al. 2011).

There was no significant difference in deformity prevalence between diploid and triploid brown trout in the present study. This is in contrast to many studies in salmonids that have reported increased prevalence of morphological deformities in triploid salmonid, leading to lower survival rates and/or growth during early development (Benfey 1999; Preston et al. 2013; Fraser et al. 2013). In this study, diploid and triploid trout displayed a low detectable prevalence of vertebrae deformities (diploid 0.7 %, triploid 1.0 %). Interestingly, the type and location of deformity differed between ploidy, with 73 % of deformity associated with decreased intervertebral space in diploids principally located within the tail region, while triploids displayed a wider range of deformity type including decreased intervertebral space, compression and fusion, representing 88 % of total deformity observed. Deformity was principally located within the cranial trunk and caudal trunk areas in triploid trout as opposed to the tail region observed in diploids (Fjelldal and Hansen 2010). The prevalence of deformities reduces the aesthetic and commercial value of the fish, but could also affect swimming ability and subsequently the fighting qualities of a sport fish (Powell et al. 2009). Indeed, most studies indicate that the causal mechanisms for deformity prevalence in triploids are physical (Fjelldal and Hansen 2010; Leclercq et al. 2011) or dietary related (Fjelldal et al. 2015). Present results suggest that deformity prevalence in diploid and triploid brown trout may be less of a concern compared to other salmonids, e.g. Atlantic salmon (Fjelldal and Hansen 2010).

The results of this study showed that diploid and triploid brown trout have similar feed intake profiles at lower temperature; however, when exposed to elevated temperature, triploid trout feed intake was significantly higher than diploids. The plasma metabolites response to exhaustive exercise of triploids was comparable to diploids although clear temperature-dependent effects were found. This suggests that triploids may not take longer to recover from exercise than diploids at higher temperature after fisheries capture. These findings have implications for the management of freshwater sport fisheries and indicate that triploid trout may provide freshwater fisheries with an alternative to stocking diploid trout, therefore reducing the negative impacts associated with stocking diploid brown trout.
